# Evaluating the Scale-Up of Antiretroviral Treatment Sites in Kwazulu-Natal Province of South Africa: Achievements and Challenges from 2005 to 2010

**DOI:** 10.5539/gjhs.v6n3p104

**Published:** 2014-02-22

**Authors:** Malangu N

**Affiliations:** 1 School of Public Health, Medunsa Campus, University of Limpopo, South Africa

**Keywords:** antiretroviral treatment, evaluation, scale-up, South Africa

## Abstract

In order to provide care to the increasing number of people infected with HIV, there is a need for scaling up the number of treatment sites. For the public health officials and planners, there is a need for a defined methodology to do this, taking into consideration the national targets as enacted by the National Department of Health (NDOH) of South Africa. This commentary is about an evaluation conducted to review the progress made by the Province of KwaZulu-Natal in scaling up antiretroviral treatment sites (ART). Based on a mathematical modelling combined with a geographical information system by Wilson and Blower, the prediction that 54 ART facilities were required for equitable distribution of antiretroviral treatment in KwaZulu-Natal had been exceeded as 89 ART sites had been established by 2010. Despite this success, two major challenges are still lurking into the ART program, namely, the accessibility of ART by those who need it and the shortage of professional human resources particularly pharmacy staff. Innovative strategies are needed to address the shortage of health professionals and related disparities in order to increase access to ART.

## 1. Introduction

**Figure 1:**
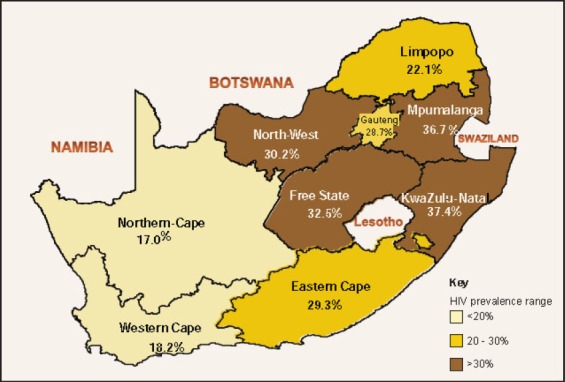
HIV Prevalence in pregnant women per province (Source: 2011 National Surveillance data)

In August 2005, we set out to use the Wilson-Blower Method to determine the number of antiretroviral treatment sites per district in the province of KwaZulu-Natal Department of Health (KZN-DOH). Our assessment took into account the population served and the number of existing sites. In doing so, we highlighted the gaps (Malangu, 2005). The particular interest in Kwazulu-Natal stemmed from the fact that this province is one of the most affected provinces in South Africa. Although the prevalence is seemingly decreasing, it is still higher in comparison to other provinces (Figure 1). Between 2009 and 2011, overall, the HIV prevalence among pregnant women decreased from 39.5% to 37.4%; while in teenagers, it decreased from 22% to 16.8%; and from 37.2% to 33.3% among young adult women aged 20 to 24 years old (National Department of Health, 2012). Moreover, there is also a huge disparity across districts within the same province; the highest prevalences of HIV in pregnant women were recorded in two districts, Ugu (41.7%) and Mkhanyakude (41.1%) (National Department of Health, 2012).

Wilson and Blower (2005) developed a mathematical model that could inform policymakers’ decisions regarding the optimal distribution of treatment sites to ensure equal access by all individuals infected with HIV. Applying this tool to the province of KwaZulu–Natal, Wilson and Blower were able to estimate that 54 ART sites would lead to the greatest fairness in the geographical distribution of ART. Subsequently, Malangu (2005) using the above finding and taking into account the population per district, the estimated 54 ART sites were apportioned to each district.

This commentary is about an evaluation conducted to review the progress made by the Province of KwaZulu-Natal in scaling up antiretroviral treatment sites (ART). Additionally, we explore the challenges of human resources associated with the scaling-up of ART sites. In doing so, it is hoped that other decision-makers could learn from the experiences from KwaZulu-Natal as they expand ART services to their patients.

## 2. Methods

The main document reviewed for this paper was the 2010 Annual Report from the Department of Health of Kwazulu-Natal province. Other relevant literature was also consulted as well as some data from the 2013 Annual Reports of Kwazulu-Natal and Gauteng provinces. Based on data from the 2010 Annual Report of Kwazulu-Natal province, the findings are summarized in Tables 1 to 3.

**Table 1:** Number of Antiretroviral Treatment Sites and patients by December 2010

District	Actual # sites by 2010	# sites predicted	Status by 2010	# patients registered	AVG # patients per site
DC-21-Ugu	4	4	Achieved	32,005	8001
DC-22-Umgungundlovu	11	5	Exceeded by 2	41,126	3739
DC-23-Uthukela	3	4	Short of 1	26,722	8907
DC-24-Umzinyathi	4	3	Exceeded by 1	15,984	3996
DC-25-Amajuba	4	3	Exceeded by 1	16,557	4139
DC-26-Zululand	9	5	Exceeded by 4	22,910	2546
DC-27-Umkhanyakude	5	3	Exceeded by 2	34,302	6860
DC-28-Uthungulu	10	5	Exceeded by 5	37,437	3744
DC-29-iLembe	8	3	Exceeded by 5	20,576	2572
DC-43-Sisonke	7	2	Exceeded by 5	15,076	2154
Durban-eThekwini	24	17	Exceeded by 7	77,861	3244
					
**Total**	**89**	**54**	**Exceeded by 35**	**340,556**	**Average=AVG= 4537**

Legend: # = number

**Table 2:** Population size per district

District	% Population	# Population	# sites by 2010	% sites	% difference
DC-21-Ugu	7.0	711,117	4	4.5	-2.5
DC-22-Umgungundlovu	9.0	914,294	11	12.4	3.4
DC-23-Uthukela	7.0	711,117	3	3.4	-3.6
DC-24-Umzinyathi	5.0	507,941	4	4.5	-0.5
DC-25-Amajuba	4.0	406,353	4	4.5	0.5
DC-26-Zululand	9.0	914,294	9	10.1	1.1
DC-27-Umkhanyakude	6.0	609,529	5	5.6	-0.4
DC-28-Uthungulu	9.0	914,294	10	11.2	2.2
DC-29-iLembe	5.0	507,941	8	9.0	4.0
DC-43-Sisonke	5.0	507,941	7	7.9	2.9
Durban-eThekwini	34.0	3,453,999	24	27.0	-7.0
					
**Total**	**100.0**	**10,158,820**	**89**	**100.0**	

Legend: # = number

**Table 3:** Number of Professional staff at ART sites

District	Expected # professionals per district			Actual # posted professionals per district			Actual # posted professionals per site		
	Medical Officers	Professional Nurses	Pharmacists Medical	Officers	Professional Nurses	Pharmacists	Medical Officers	Professional Nurses	Pharmacists
DC-21-Ugu	64	128	64	37	95	15	9	24	4
DC-22-Umgungundlovu	82	165	82	48	122	19	4	11	2
DC-23-Uthukela	53	107	53	31	79	13	10	26	4
DC-24-Umzinyathi	32	64	32	19	48	8	5	12	2
DC-25-Amajuba	33	66	33	19	49	8	5	12	2
DC-26-Zululand	46	92	46	27	68	11	3	8	1
DC-27-Umkhanyakude	69	137	69	40	102	16	8	20	3
DC-28-Uthungulu	75	150	75	44	111	18	4	11	2
DC-29-iLembe	41	82	41	24	61	10	3	8	1
DC-43-Sisonke	30	60	30	18	45	7	3	6	1
Durban-eThekwini	156	311	156	91	231	37	4	10	2

Legend: # = number

## 3. Results

Overall, the prediction that 54 ART facilities were required for equitable distribution of antiretroviral treatment has been exceeded. Based on the calculations performed in 2005, one district, Ugu, achieved the number predicted; and another, Uthukela was short of one facility as shown in Table 2. The remaining districts exceeded the target by setting up one to seven more ART facilities.

By the end of 2010, the average number of patients was 4,537 per site; however, in three districts, namely, Umkhanyakude, Ugu and Uthukela, there were more than the average number of patients per facility. As shown, in Table 2, these three districts as well as the Ethekwini district had less than the required number of sites based on the populations in their catchments.

These disparities were such that Ugu and Uthukela districts which hold each 7% of the population had respectively 4.5% and 3.4% of ART sites. Similarly, Ethekwini district that has 34% of the population, held 27% of ART sites.

Overall, by 2010, 89 ART sites had been established. Recent data show that the number of facilities providing ART services increased from 89 ART sites in 2008 to 608 by mid-2013. The total number of patients on ART increased from 225,389 patients in 2008 to 726,338 patients by mid-2013, with about 178,927 new patients having been initiated on ART during 2012 (Kwazulu-Natal Department of Health ans Social Welfare, 2013).

Another positive finding was that there was a decrease in the number of patients lost to follow-up. There were 6.3% (21.541.0 out of 340,556.0 registered patients) by 2010, but there are now 4.1% (29,477.0 out of 726,338 registered patients; of this number, 7,386.0 patients or 1% of them being reported dead. This increase is due to the introduction of a new initiative called “Nurse Initiated and Managed ART (NIMART)”; since 2010, a total of 1,578 nurses have been trained on NIMART and certified to prescribe ART (KZN-DOH, 2013).

## 4. Discussion

The above findings suggest two things, firstly that the Wilson-Blower model was useful in predicting the initial number of sites required but the effect of the scaling-up of antiretroviral treatment had been missed. Secondly, although the predicted number of sites was exceeded, there is still need for more sites for two reasons.

The first reason is that the provincial department of Health of Kwazulu-Natal has not yet met the national target for ART coverage. It is reported that 60% of people who need ART in this province, actually receive it; this is well below the national target of 80% (KZN-DOH, 2010; NDOH, 2003).

The second reason is the shortage of human resources coupled to its maldistribution in this large province; it seems that an average of 4537 patients per site is still too high when the number of professional staff members is taken into account as explained below. For most remote and rural areas, there is a need for sites that are closer to them. For instance, in comparison to Gauteng Province, ART is available from 90.3% of all public health facilities. There are 5,582 nurses that have been certified to initiate and manage ART in Gauteng, almost three times the corresponding figure for Kwazulu-Natal as stated above (Gauteng-DOH, 2013).

With regard to the targets set for human resources, nationally, the optimal staff complement needed to deliver ART was set as 1 medical officer; 2 professional nurses; 1 pharmacist; 1 dietician/nutritionist; a part-time (50%) social worker, per 500 patients (NDOH, 2003). Hence, based on the average of 4537 patients per site (Table 1), one would have expected 9 medical officers, 9 pharmacists and 18 professional nurses per site. As shown in Table 3, none of the districts met the target for pharmacists; however, Ugu, Uthukela, and Umkhanyakude exceeded the target for professional nurses; Ugu met the target for medical officers, while Uthukela exceeded it (26 instead of 18).

Furthermore, the national targets were still not met based on the number of registered patients and the number of posts filled in; the vacancy rates are high and have been estimatd to be as high as 25.7% for professional nurses, 41.6% for medical doctors, and 76.4% for pharmacist (KZN-DOH, 2010). Hence based on the number of existing staff members, it is can be calculated that, on average, the actual staff complement in 2010 was one professional nurse for 336 patients instead of 250; 1 medical officer for 856 patients instead of 500; and one pharmacist for 2119 patients instead of 500. Thes figures show that the shortage of pharmacists is more severe than that of medical officers and nurses (Table 3). It is clear that filling the posts for pharmacists represents one of the major challenges in the near future. This situation calls for a redefinition of pharmacy staffing norms: should the number of pharmacists continue to be used as a target or rather the number of pharmacist’ assistants?

It is my opinion that a revised target should rather be set for five pharmacists’ assistants per site with 4500 patients. This is based on 20 working days per month on which ART patients are served, 225 patients per day (4500/20), 10 minutes spent per patient, 48 prescriptions dispensed (eight working hours=480minutes/10) per assistant per day. Since it is faster and even cheaper to train pharmacists’assistants than pharmacists, intensifying the training program for pharmacist’assistants could assist in addressing the shortage of pharmacy personnel required to do the dispensing of antiretroviral and other medicines.

Finally, further research is needed to establish whether the suggested staffing targets correlate with the actual workload and how satisfied, staff members and patients are (MSH and WHO, 2006; van Rensburg et al., 2008; Mayosi et al., 2011).

In conclusion, the predicted number of ART sites by Wilson and Blower method was largely exceeded within 5 years of their prediction. Two major challenges still lurking into the ART program were the accessibility of ART by those who need it and the shortage of professional human resources particularly pharmacy staff. Innovative strategies are needed to address the shortage of health professionals and related disparities in order to increase access to ART.
